# P-921. A medication use evaluation of cefiderocol for the treatment of multi-drug-resistant gram-negative infections at a tertiary care medical center: a healthcare/quality improvement project

**DOI:** 10.1093/ofid/ofaf695.1127

**Published:** 2026-01-11

**Authors:** Ogenetega Madedor, Cassidy Boomsma, Alok Nimgaonkar, Lan Duong, Princy N Kumar, Semithe Chevelon, Joseph G Timpone

**Affiliations:** MedStar Georgetown University Hospital, Washington, District of Columbia; Medstar Georgetown University Hospital, Washington, DC; Medstar Georgetown University Hospital, Washington, DC; Medstar Georgetown University Hospital, Washington, DC; Georgetown University Medical Center, Washington, District of Columbia; St. George's University, LAUDERDALE LAKES, Florida; Medstar Georgetown University Hospital, Washington, DC

## Abstract

**Background:**

Cefiderocol (CFDC) is a novel advanced cephalosporin with broad activity against gram-negative bacilli, including those with multi-drug resistance. It acts as a siderophore, binding to extracellular iron which then facilitates its transport across the gram-negative cell membrane. There has been a steady rise in antimicrobial resistance at our institution, infections caused by carbapenem-resistant (CR) pathogens, and therefore an increased need for advanced generation agents such as CFDC.

**Table 1:**
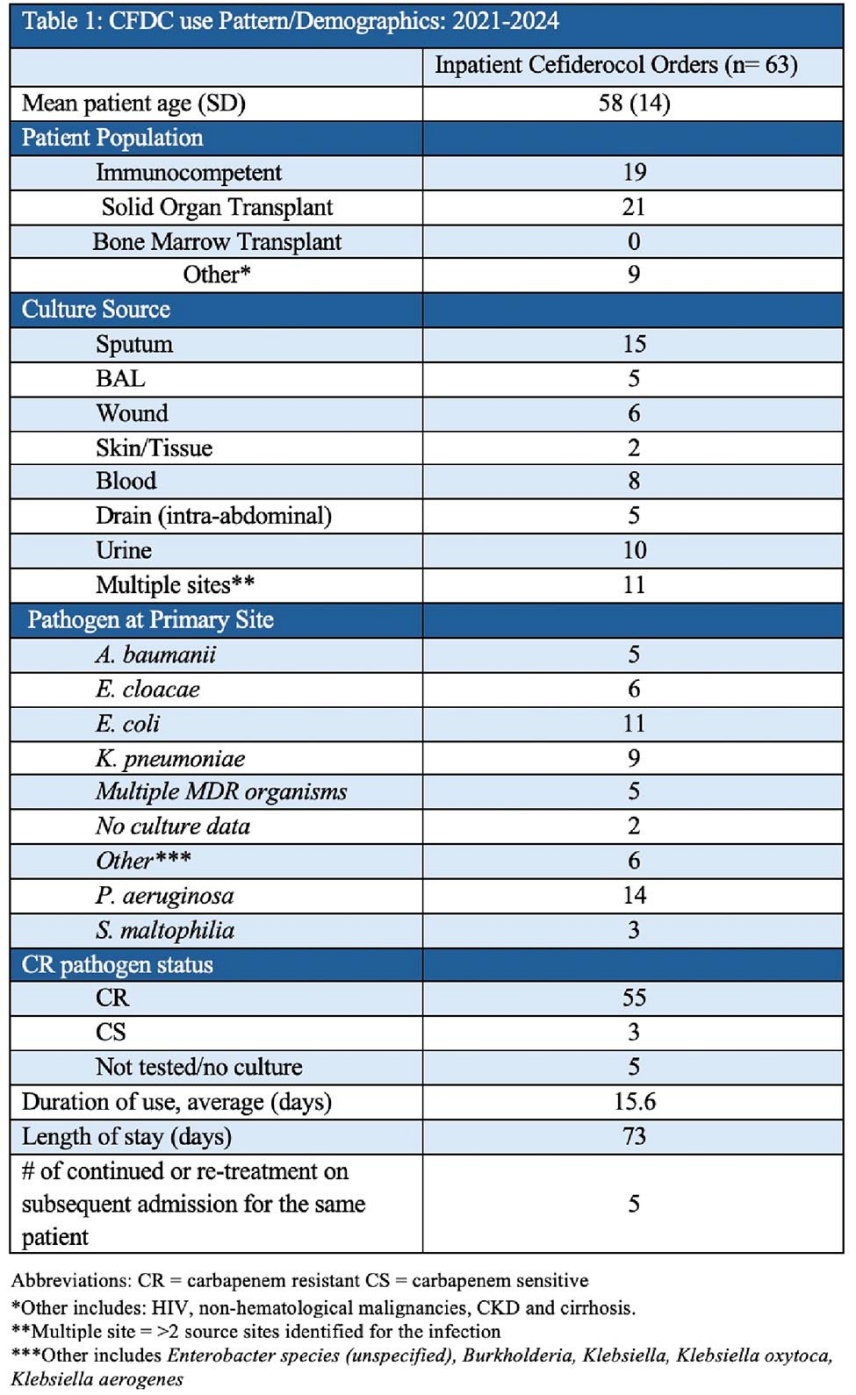
CFDC use pattern/demographics: 2021-2024

**Table 2:**
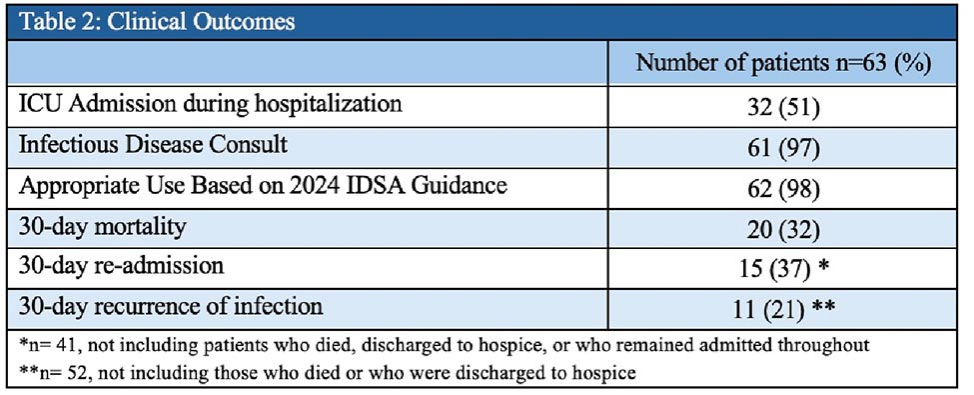
Clinical Outcomes

**Methods:**

We performed a single center retrospective chart review of CFDC use for resistant gram-negative infections from 2021-2024. Patients met inclusion criteria if they received CFDC for definitive management of clinical infection based on antimicrobial susceptibilities. Clinical characteristics were identified including pathogens, sites of infection, duration of therapy and patient outcomes including 30-day overall mortality. Appropriate use of CFDC was defined based on IDSA’s *2024 Guidance on the Treatment of Antimicrobial Resistant Gram-Negative Infections* in addition to our local antibiotic stewardship guidelines.

**Table 3:**
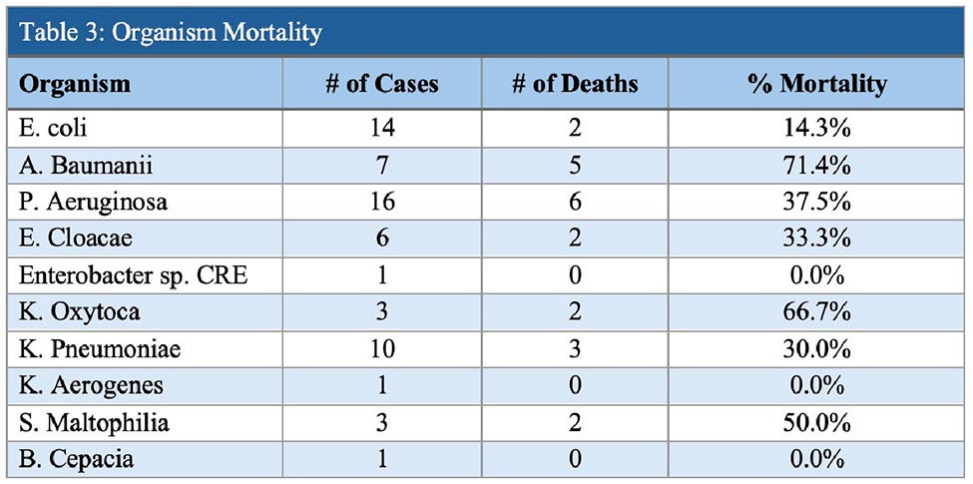
Organism Mortality

**Table 4:**
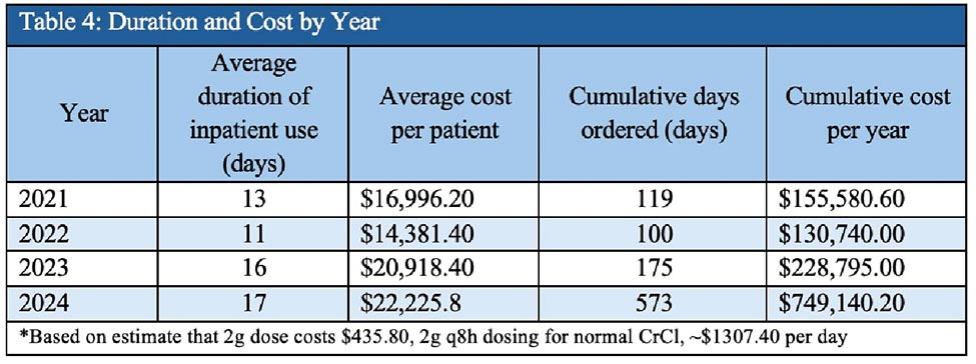
Duration and Cost by Year

**Results:**

63 patients with an average age of 58 were treated from 2021– 2024 with CFDC for a Mean Duration of 15.6 days and length of stay of 73 days. We identified 55 CR isolates; E. coli and P. aeruginosa accounting for >45% (Table: 3). ID consultation was obtained in 96.8% (61/63) orders of CFDC, with 52% of those patients requiring an ICU escalation due to respiratory failure or hemodynamic instability. Overall mortality was found to be 32%, with the highest rates occurring in Acinetobacter baumannii (71%; 5/7) (Table 3). Appropriate use of CFDC as defined by IDSA guidance was seen in 98.4% (62/63) of patients. Cumulative Hospital CFDC costs per year increased from $155,580 in 2021 to $749,140 in 2024 (Table: 4).

**Conclusion:**

Despite good adherence to IDSA guidelines, CFDC usage has increased at our institution, and this parallels the rise in CR infections. The treatment of CR infections with CFDC has resulted in a significant financial burden to our institution.

**Disclosures:**

All Authors: No reported disclosures

